# Remote sensing for monitoring and managing *Eichhornia crassipes*: a review of methods, applications, and decision-support frameworks

**DOI:** 10.1007/s10661-026-15788-y

**Published:** 2026-09-02

**Authors:** Daniel H. C. Salim, Caio C. S. Mello, Gabriel Pereira, Raian V. Maretto, Camila C. Amorim

**Affiliations:** 1 Department of Sanitary and Environmental Engineering, Universidade Federal de Minas Gerais, Belo Horizonte, Minas Gerais Brazil; 2 Department of Geosciences, Universidade Federal de São João del-Rei, São João del-Rei, Minas Gerais Brazil; 3 Faculty of Geo-Information Science and Earth Observation, University of Twente, Enschede, the Netherlands

**Keywords:** Aquatic invasive plants, Freshwater ecosystems, Multi-sensor integration, Species discrimination, Adaptive management, Early-warning indicators

## Abstract

*Eichhornia crassipes* (water hyacinth) is among the world’s most aggressive aquatic invasive plants, with severe ecological and socioeconomic impacts on freshwater ecosystems. Over the last two decades, remote sensing has emerged as a critical tool for monitoring its spread and supporting management decisions, offering scalable, repeatable, and increasingly precise assessments. This review synthesizes 56 studies published between 2004 and 2025, examining how different platforms (satellites, UAVs, airborne, proximal), sensor types (multispectral, hyperspectral, SAR), and analytical methods (index thresholding, object-based classification, machine learning, deep learning) have been applied to detect, classify, and quantify *E. crassipes*. We highlight the evolution from early single-platform approaches to recent multimodal frameworks that integrate optical, radar, and UAV data, enabling both large-scale surveillance and fine-resolution diagnostics. Evidence demonstrates that species-level discrimination, phenological tracking, and biomass estimation are now feasible, with promising developments in linking spectral responses to pollutants and stress indicators. Despite these advances, key challenges remain in harmonizing data across platforms, reducing misclassification in mixed vegetation assemblages, and translating remote sensing outputs into decision-relevant information for adaptive management. We propose a decision-support framework built on five strategic pillars: multi-scale sensor fusion, ecologically interpretable classification schemes, scalable analytical ladders, early-warning indicators, and feedback loops linking monitoring to management actions. Together, these directions show how remote sensing can move beyond detection toward more responsive and decision-oriented approaches that support the sustainable management of *E. crassipes* invasions across diverse aquatic environments.

## Introduction

Originating from the Amazon Basin, *Eichhornia crassipes* has become one of the most aggressive invasive species globally, especially in tropical and subtropical freshwater ecosystems (Mahamood et al., 2023). Since its introduction outside its native habitat in the 1800 s, the plant has proliferated quickly, causing significant environmental and socioeconomic challenges across numerous countries (Aqdas & Hashmi, 2023). Its ability to thrive in nutrient-rich waters and form dense mats (Amalina et al., 2022), with biomass densities exceeding 60 kg per square meter (Wilson et al., 2005), led the International Union for Conservation of Nature (IUCN) to classify it among the world’s 100 most invasive species (Lowe et al., 2000).

*Eichhornia crassipes* has also been recognized for its potential role as a bioindicator of water pollution, such as endocrine disruptors and neonicotinoids (De Laet et al., 2019) and for assessing heavy metal contamination and toxicity, making it useful for environmental risk assessments (Mahamood et al., 2023). In addition, studies have shown that *E. crassipes*, when analyzed alongside water quality measures, serves as a reliable indicator of pollution, including nutrients and chemical contaminants (Tabrez et al., 2022). This dual nature, both invasive and diagnostic, is responsible for *E. crassipes* being a relevant research field related to ecological and environmental fields.

The environmental impact of *E. crassipes* is multifaceted, with its ability to form thick mats on water surfaces leading to detrimental effects on aquatic ecosystems. These mats reduce sunlight penetration, severely affecting the photosynthetic process in aquatic plants and leading to oxygen depletion in water bodies (Wu & Ding, 2020). As a result, water quality deteriorates, and the balance of aquatic communities is disrupted (Hestir et al., 2008; Mouta et al., 2023). Studies show that the presence of *E. crassipes* initially enhances habitat complexity, attracting aquatic invertebrates, but as its dominance increases, the diversity and abundance of native species decline (Villamagna & Murphy, 2010). This ecological imbalance not only threatens biodiversity but also has severe socioeconomic repercussions. The obstruction of waterways affects transportation, fishing, and access to clean water (Basaula et al., 2023; Djihouessi et al., 2023; Ongore et al., 2018). Moreover, the substantial costs incurred in controlling and eradicating *E. crassipes* infestations significantly impact local economies (Kleinschroth et al., 2021; May et al., 2022; Yan et al., 2017).

*E. crassipes* is a free-floating macrophyte that forms dense rosettes with buoyant, aerenchyma-rich petioles and fibrous submerged roots. This morphology favors rapid canopy formation over slow-moving, nutrient-enriched waters and a wide range of conditions (temperate to tropical, variable pH, pollutants) (El-Gendy et al., 2004; Reddy et al., 1989; Rezania et al., 2015). Its clonal growth via stolons yields very fast doubling times, and long-lived seed banks sustain reinvasion pulses after control (Malik, 2007; Ouma et al., 2005). These traits produce large, spectrally distinctive floating mats that are well captured by optical red-edge/NIR features and, under certain configurations, and by radar backscatter contrasts, directly motivating the multi-sensor strategies reviewed here.

Due to its spatial extension, remote sensing has emerged as a powerful tool for monitoring and managing the spread of *E. crassipes*. Traditional ground-based monitoring methods, while useful, are often limited in scale and can be labor-intensive, time-consuming, and low-efficiency (Hestir et al., 2008; Zhang et al., 2019). Remote sensing methods provide large-scale, continuous monitoring capabilities that can track the extent and diagnose the *E. crassipes* invasions over water bodies (Ghoussein et al., 2019; Kleinschroth et al., 2021). The ability to detect and monitor these plants using various spectral bands, such as optical hyperspectral and multispectral, and microwave sensors, allows researchers to observe the spatial distribution and health status of *E. crassipes* across the water bodies, enabling timely interventions for management and control.

**Fig. 1:**
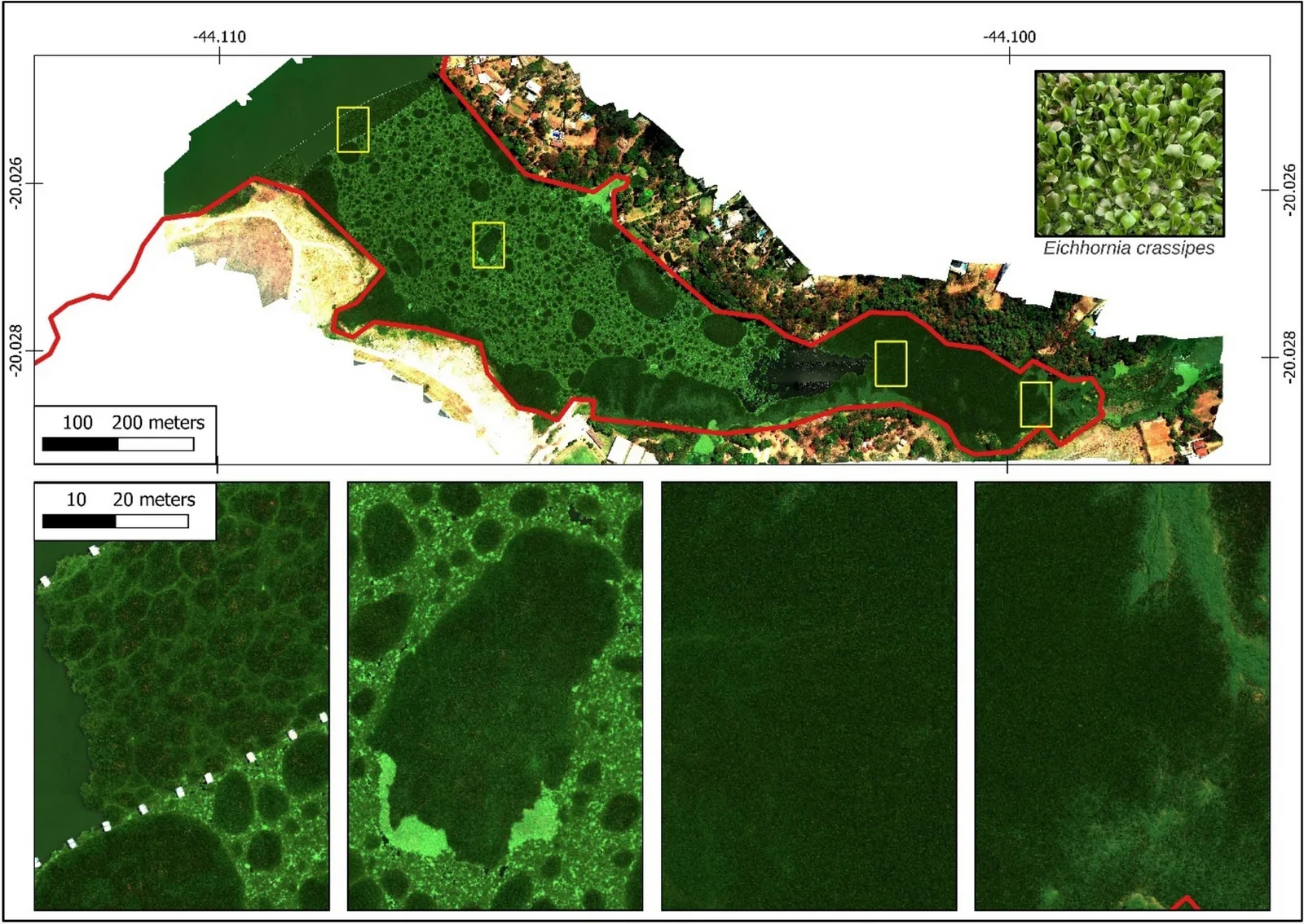
UAV orthomosaic produced by MicaSense RedEdge-P dual imagery (6 cm/px) over a section of the Ibirité Reservoir, Brazil, depicting extensive infestation by aquatic plants, primarily *Eichhornia crassipes*. The high spatial resolution of UAV data enables detailed visualization of infestation patterns that cannot be resolved by satellite imagery. Insets below illustrate this advantage by revealing (left to right): *E. crassipes* enclosed by a protective net; several small mats of *E. crassipes* adjacent to a large mat with *Pistia stratiotes* entangled; a homogeneous mat of *E. crassipes*; and a mixed mat containing *Brachiaria subquadripara*

A major advancement in remote sensing technology for detecting *E. crassipes* is the development of multi-platform observations that integrate multiple data sources. Combining satellites, UAVs, MAVs, and ground-based sensors allows for comprehensive spatial and temporal analysis, providing a more reliable dataset for long-term environmental monitoring. For instance, Synthetic Aperture Radar (SAR) technology offers an all-weather advantage, which is particularly valuable in regions and seasons with frequent cloud cover (DeVries et al., 2020; Torres et al., 2012). UAV optical sensors, on the other hand, deliver high-resolution spectral data during clear weather, facilitating detailed analysis of plant health and initial spread. The synergy of these platforms has revolutionized the detection and management of invasive aquatic plants, enabling more timely and cost-effective interventions (Bolch et al., 2021; Pádua et al., 2022a, 2022b; Simpson et al., 2022). This integrated approach is exemplified in Fig. 1, which shows a high-resolution UAV orthomosaic that captures the fine-scale spatial heterogeneity and mixed species composition of aquatic plant infestations, dominated by *Eichhornia crassipes*, in a reservoir in Brazil.

Although previous reviews have advanced the understanding of *Eichhornia crassipes* monitoring, they reveal important limitations in scope, methodological depth, and technological integration (Aguiar & Ferreira, 2013; Datta et al., 2021; Thamaga & Dube, 2018a; Vaz et al., 2018). Over the past two decades, remote sensing technologies have evolved rapidly, enhancing the ability to detect and monitor aquatic vegetation with greater spatial, temporal, and spectral detail. In response to these advances, there is a need for a more comprehensive and analytically focused review that critically evaluates the role of remote sensing in supporting the detection, monitoring, and management of *E. crassipes*.

This review addresses this gap by mapping the methodological landscape for detecting and monitoring *Eichhornia crassipes* across remote sensing platforms (satellite, airborne, UAV, proximal) and sensor types (multispectral, hyperspectral, SAR), while synthesizing RS analytical approaches and reported performance metrics across freshwater ecosystems contexts. We evaluate the added value of multi-sensor integration, particularly optical–SAR fusion and UAV–satellite complementarity, and assess current capabilities in species discrimination, phenological tracking, biomass estimation, and spectral proxies of stress and pollutants. By linking remotely sensed dynamics to ecological drivers and management actions, we outline a path toward more informed and responsive monitoring strategies. Finally, we propose an operational framework grounded in five pillars: (i) multi-scale sensor fusion; (ii) ecologically interpretable class schemes; (iii) a scalable analytical ladder; (iv) early-warning indicators; and (v) adaptive feedback loops connecting monitoring to intervention.

## Methodology

This study presents a literature review of remote-sensing applications for *Eichhornia crassipes* in natural and managed freshwater systems. The literature search and study selection process were structured and reported in accordance with the PRISMA 2020 framework (Page et al., 2021). We synthesized how platforms (satellite, airborne/manned aircraft, UAV, and proximal/handheld), sensor types (RGB, multispectral, hyperspectral, and SAR), and analytical approaches (thresholding, object-based image analysis, machine learning, and deep learning) have been used to detect, map, monitor, and quantify the species. Beyond cataloging technologies, we examined full processing pipelines, including image acquisition, preprocessing, feature engineering, and model training, validation, and reporting, to compare methodological choices across environmental settings and operational constraints.

The search strategy combined the species term *Eichhornia crassipes* with platform and sensor keywords (remote sensing; satellite; UAV/UAS/drone; multispectral; hyperspectral; synthetic aperture radar), using Boolean operators applied to titles, abstracts, and keywords in Scopus, IEEE and in Google Scholar. All records were exported, deduplicated, and consolidated prior to data analysis. Studies employing GIS-based applications and spatial analytical approaches were eligible when they used remote-sensing data to elucidate *E. crassipes* dynamics.

The search yielded 128 documents; after removing duplicates and erroneous entries, 67 were excluded because they lacked a DOI or traceable record, were not in English, or did not contain relevant use of remote-sensing data for *E. crassipes*. This criterion may have excluded relevant regional or gray literature, but was adopted to ensure traceability and reproducibility. The final selection comprised 61 documents—57 original research articles and four reviews. Review articles informed background and thematic triangulation but were excluded from quantitative accuracy summaries. Additionally, multi-species studies in which *E. crassipes* was peripheral, underrepresented, or not adequately analyzed were retained for qualitative synthesis only and excluded from all quantitative analyses.

From each study we extracted: bibliographic details; study location and waterbody type; platform(s); sensor(s); application type; preprocessing steps (e.g., atmospheric correction, speckle filtering, orthomosaic generation); features/indices (e.g., normalized difference vegetation index (NDVI), red-edge indices, normalized difference chlorophyll index (NDCI), texture measures, polarimetric metrics); algorithm(s) (thresholding, object-based, machine learning, deep learning); training/validation design and metrics (overall accuracy, producer’s/user’s accuracy, F1, IoU); ground-truthing approach and temporal alignment; and evidence of platform/sensor integration. Reported accuracy was summarized as counts and proportions to emphasize typical ranges across heterogeneous metrics and validation designs; no formal meta-analysis or bibliometric analysis was undertaken. Studies were grouped by platform, sensor type, and algorithm family, while considering environmental context (waterbody type, season/acquisition conditions) and integration strategy (single vs multi-platform/sensor, including optical–SAR fusion and UAV–satellite complementarity).

## Remote sensing platforms: detecting *E. crassipes* from the ground to satellites

**Fig. 2:**
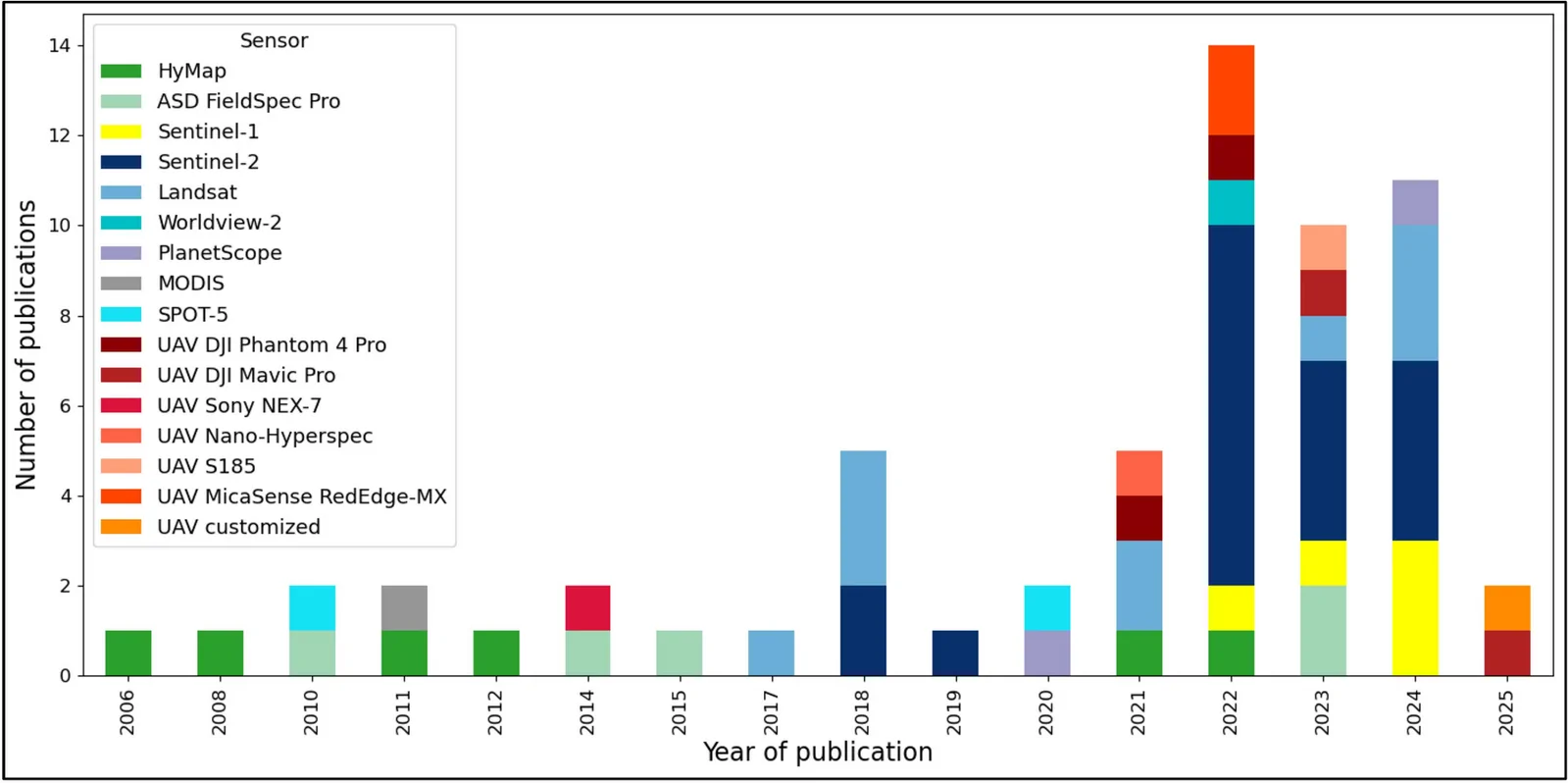
Annual distribution of publications by remote sensing sensor type (2006–2025). Publications on *Eichhornia crassipes* monitoring show a steady increase, with a marked rise after 2020. Sentinel-2 rapidly became the most widely used satellite platform, while Sentinel-1 gained importance for its all-weather capability and UAVs for their high-resolution monitoring at local scales

**Fig. 3:**
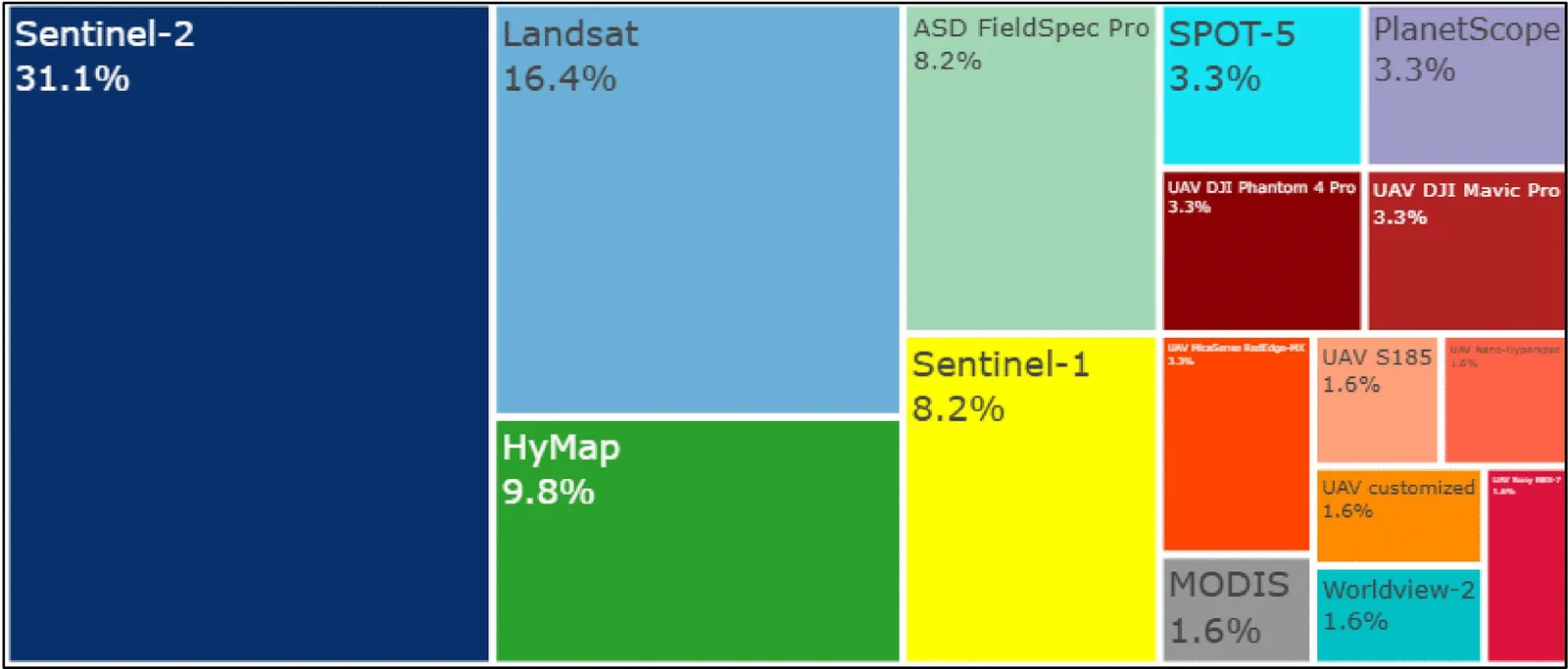
Relative contribution of remote sensing platforms in studies of *Eichhornia crassipes* (2006–2025). Multispectral satellites dominate, with Sentinel-2 leading and Landsat providing long-term coverage. Hyperspectral sensors, mainly HyMap and ASD FieldSpec Pro, also play an important role in species discrimination and spectral validation. Sentinel-1 and UAV platforms account for a smaller but increasing share of contribution

The choice of sensing platform has profoundly influenced the trajectory of remote sensing applications in aquatic vegetation monitoring, particularly for *Eichhornia crassipes*. Each technological advance has expanded the spatial and temporal scales of observation while redefining what is feasible in terms of spectral resolution, revisit frequency, and operational cost. Over time, research has evolved from localized campaigns using handheld spectroradiometers, to manned airborne hyperspectral surveys, and finally to satellite constellations that provide systematic global coverage. This progression is evident in Fig. 2, which illustrates the annual distribution of publications by sensor type between 2006 and 2025, and in Fig. 3, which highlights the proportional reliance on each sensor across reviewed studies. Together, these figures underscore the methodological shift toward broader coverage and operational scalability, with Sentinel-2 rapidly emerging as the dominant platform after its launch in 2015.

The first studies on this topic emerged in the mid-2000s, during which manned airborne campaigns formed the backbone of large-scale spectral assessments. This period was exemplified by the repeated use of the HyMap hyperspectral sensor over the Sacramento–San Joaquin River Delta in California. Papers (Hestir et al., 2008; Khanna et al., 2011, 2012; Underwood et al., 2006) demonstrated the capacity of airborne hyperspectral imagery to differentiate *E. crassipes* from co-occurring aquatic vegetation and substrate features. These campaigns provided critical insights into spatial heterogeneity and seasonal phenology, but the operational complexity and high cost of manned flights limited their scalability and temporal flexibility. Despite these constraints, airborne sensors established a methodological baseline that continues to inform the interpretation of higher-altitude imagery (Hestir et al., 2008; Khanna et al., 2011, 2012; Underwood et al., 2006).

Contemporaneous with airborne campaigns, ground-based hyperspectral platforms played a vital role in physiological monitoring and spectral validation. Handheld instruments such as the ASD FieldSpec Pro FR were used to capture fine-scale reflectance data under controlled conditions. In South Africa, Newete et al. (2014) employed this technology to examine the effects of heavy metals and biological control agents on *E. crassipes*, revealing strong spectral responses in red-edge indices associated with chlorophyll content. In the USA, Robles et al. (2010, 2015) combined handheld hyperspectral data with simulations of Landsat-5 Thematic Mapper bands to evaluate herbicide efficacy and the spectral behavior of biomass. While limited to a spatial extent, these ground-based studies provided high-resolution biophysical insights and served as critical ground-truth references for airborne and satellite observations.

As sensor technologies evolved, satellite platforms became increasingly central to methodological approaches in monitoring *E. crassipes*. Early use of systems like SPOT-5 (Schmidt & Witte, 2010) and MODIS (Kiage & Obuoyo, 2011) enabled broad-scale classification and time-series analyses. However, their relatively coarse spatial resolutions limited their effectiveness in detecting small or fragmented infestations. Landsat imagery, particularly from missions 5, 7, and 8 (Kleinschroth et al., 2021; Mukarugwiro et al., 2021), gained popularity for retrospective monitoring, offering a rare combination of long-term temporal coverage (over four decades) and consistent multispectral data. A major turning point occurred in 2015 with the launch of Sentinel-2. Its superior spatial resolution (10–20 m), high revisit frequency, and inclusion of red-edge bands made it particularly well suited for regional-scale monitoring of *E. crassipes* (Bayable et al., 2023; Thamaga & Dube, 2018b). Sentinel-2 emerged as the dominant platform in recent studies, appearing in 31% of the studies reviewed (Fig. 3) and showing a marked increase in use after 2020 (Fig. 2), reflecting both its technical capabilities and broad accessibility.

**Fig. 4:**
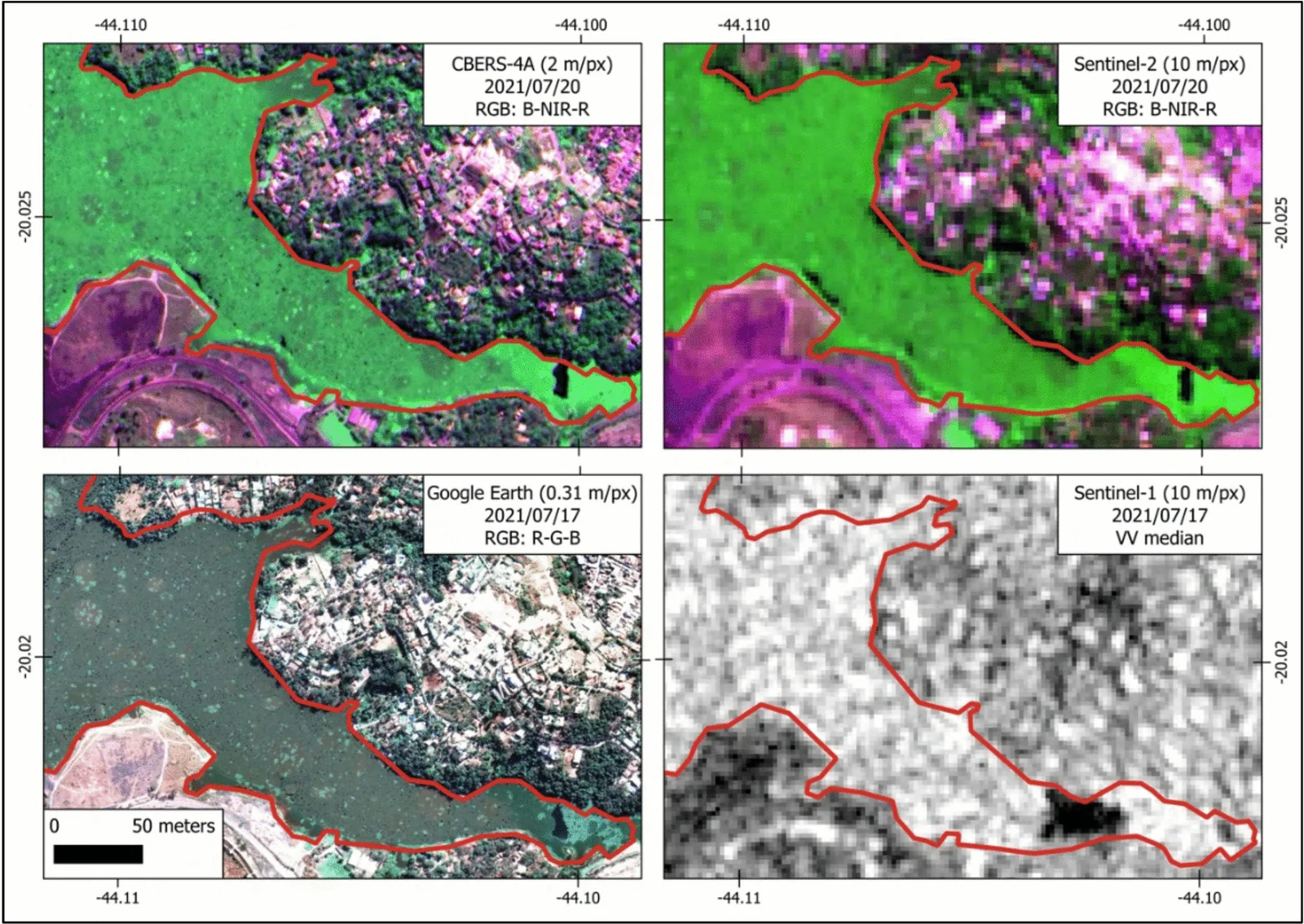
Multi-platform comparison and tradeoffs of aquatic invasive plant coverage, primarily *Eichhornia crassipes*, in the Ibirité Reservoir, Brazil (limited by the red line). Google Earth provides historic RGB imagery with the highest spatial detail, but with irregular availability. CBERS-4A, with NIR sensitivity, offers monthly acquisitions with finer spatial resolution than Sentinel-2. Sentinel-2 adds greater spectral depth for species-level discrimination, while Sentinel-1 complements optical sensors by highlighting water–vegetation contrasts under all weather conditions

This transition toward platform diversification is exemplified in Fig. 4, which presents a visual comparison of *Eichhornia crassipes* infestations captured by four distinct satellite platforms: CBERS-4A, Sentinel-2, Sentinel-1, and Worldview-3 (provided by Google Earth). The figure highlights the variation in spatial resolution, spectral characteristics, and detection capabilities across platforms, with spatial detail ranging from 0.31 to 10 m per pixel. These examples underscore the trade-offs inherent in platform selection; while coarser sensors like Sentinel-1 offer consistent all-weather monitoring through SAR, finer-resolution imagery from CBERS-4A and Google Earth allows for improved delineation of *E. crassipes* mats in complex aquatic landscapes. These images illustrate the complementary nature of satellite systems in capturing different aspects of *E. crassipes* dynamics, providing a compelling rationale for integrating multiple platforms in operational monitoring strategies.

Meanwhile, UAVs have transformed the accessibility and resolution of remote sensing at local scales. Introduced into *E. crassipes* studies in 2014, UAVs initially employed consumer-grade RGB and multispectral cameras. More recently, the integration of hyperspectral scientific-grade sensors such as the Nano-Hyperspec (Bolch et al., 2021) and S185 (Mu et al., 2023) has enabled detection of *E. crassipes* among complex aquatic vegetation communities. The rapid increase of UAV-based studies post-2018, as seen in the temporal distribution of publications (Fig. 2), underscores the growing recognition of UAVs as a flexible and cost-efficient complement to satellite systems. Although still underrepresented in the literature in relative terms (Fig. 3), UAVs are poised to play an increasingly central role, particularly in multi-platform monitoring strategies aiming at small patches and initial infestations.

This platform evolution reveals a clear trajectory: from labor-intensive, high-resolution airborne surveys to scalable, multi-sensor approaches that integrate UAV agility, ground-truthing precision, and satellite breadth. The current landscape is increasingly defined by convergence, where UAV and satellite data are synchronized to overcome the limitations of any single platform. This shift not only enhances the reliability of aquatic vegetation assessments but also supports the development of operational monitoring systems that are temporally responsive, spatially adaptive, and globally scalable.

## Hyperspectral, multispectral, and SAR methods for detecting *E. crassipes*

Spectral methods in remote sensing play an important role in the detection and monitoring of *Eichhornia crassipes*, leveraging the plant’s unique reflectance properties across different spectral regions. By utilizing multispectral, hyperspectral, and microwave sensors, researchers can capture detailed information about the characteristics of *E. crassipes*. These spectral approaches offer varying advantages in terms of spatial resolution, temporal frequency, and environmental adaptability, providing critical insights for ecological assessment and management strategies. This section reviews and critiques the application of these spectral modalities in recent literature, highlighting their strengths, limitations, and contributions to the understanding of aquatic plant invasions.

Hyperspectral remote sensing provides detailed spectral information by capturing reflectance across hundreds of narrow and contiguous bands, allowing fine discrimination of vegetation biochemistry and structure. In the context of *Eichhornia crassipes* monitoring, recent studies have strategically leveraged these capabilities to enhance detection accuracy and interpret ecological dynamics. Ghoussein et al. (2023) employed a handheld ASD FieldSpec Pro FR to analyze *Eichhornia crassipes* under varying phenological stages. The study focused on red-edge and near-infrared (NIR) wavelengths, simulating Sentinel-2 bands (B6–B9) and extracting spectral slopes and vegetation indices to improve classification in mixed vegetation zones. Results showed that slope-based metrics and red-edge indices outperformed basic reflectance values, enabling the detection of physiological variation among large green, yellowing, and flowering *Eichhornia crassipes* individuals. The capacity of hyperspectral configurations to resolve subtle spectral shifts across growth stages demonstrated their value in settings with high species and phenological diversity.

Studies using UAV- and MAV-mounted hyperspectral sensors have pushed these spectral capabilities further into high-resolution spatial domains. Bolch et al. (2021) utilized the Nano-Hyperspec sensor aboard a UAV platform, capturing narrow-band spectral data across the visible to shortwave infrared (SWIR) range. Using a decision tree model, this configuration enabled accurate classification of *Eichhornia crassipes* amidst several structurally and spectrally similar emergent vegetation, such as *Pontederia cordata* and *Trapa natans*. The main indices to separate aquatic plants were Average reflectance in the near-infrared index (ARNI) and spectral reflectance change rate (SC) with 780 and 900 nm. Moreover, Mu et al. (2023), using the S185 sensor, emphasized other indices tailored to aquatic systems, such as the Chlorophyll Spectral Index (CSI), Phycocyanin Spectral Index (PSI), and Macrophyte Spectral Index (MSI). These indices targeted functional traits like chlorophyll concentration and pigment-specific absorption, enhancing spectral sensitivity to aquatic macrophytes. However, overlapping spectra among invasive aquatic plant species and environmental variability (e.g., turbidity, water bloom) reduced specificity, reinforcing the need for targeted spectral index development. Furthermore, Ade et al. (2022) demonstrated the benefit of spectral synergy by integrating HyMap with Sentinel-2 multispectral imagery. The broader spectral base allowed improved separation of *E. crassipes* and *Ludwigia* spp., particularly through the use of red-edge and SWIR bands, where Sentinel-2 alone showed reduced performance due to band coarseness and spectral overlap.

These studies demonstrate that the utility of hyperspectral configurations lies not only in spectral density but also in the careful selection of wavelengths and indices aligned with plant biochemistry and structural traits. Table 1 summarizes key studies that apply hyperspectral sensors, some in combination with multispectral data, for the monitoring and classification of *E. crassipes*, highlighting the platforms used, analytical techniques, and reported accuracies.

**Table 1:** Studies applying hyperspectral sensors for *E. crassipes* monitoring

Authors	Study area	Sensor	Application	Techniques	Performance	Findings
Hestir et al. (2008)	USA, California’s Sacramento–San Joaquin River Delta	HyMap	Classification of phenological stages and other plants	Decision tree (DT) with SMA (spectral mixture analysis), SAM (spectral angle mapper), and continuum removal (CR)	OA, 78%; *E. crassipes* PA, 69%; UA, 89%	High spectral variability in *E. crassipes* due to varied phenological stages (vegetative/healthy, flowering, stressed/senescent); Spectral overlap and limited resolution reduced distinguishing *E. crassipes* from floating species; phenology-aware models enhance monitoring accuracy and operational applicability
Bolch et al. (2021)	USA, California’s Sacramento–San Joaquin River Delta	HyMap, Nano-Hyperspec	Classification of *E. crassipes* and other plants; synchronization of UAV and MAV data	Random forest (RF)	UAV Nano-Hyperspec OA, 94%; PA, 87%; UA, 100%; MAV Hymap OA, 85%; PA, 92%; UA, 90%	Chlorophyll indices and red edge bands key for detecting *E. crassipes*; UAVs are limited by small coverage, high data demands, and operational complexity; best for localized monitoring post-treatment or early invasion detection
Ade et al. (2022)	USA, California’s Sacramento–San Joaquin River Delta	HyMap, Sentinel-2	Classification of *E. crassipes* and other plants; synchronization of MAV and satellite data	RF	HMAV OA, 90–91%; PA, 86–94%; UA, 88–92%; Sentinel-2 OA, 87–90%; PA, 78–80%; UA, 85–94%	HyMap achieved higher accuracy for *E. crassipes* detection than Sentinel-2, which demonstrated the potential to supplement HyMap by filling intra-annual temporal gaps
Ghoussein et al. (2023)	Lebanon and Syria border, Al Kabir River	ASD Field Spec Pro FR, Simulated Sentinel-2	Classification of phenological stages and other plants	Principal component analysis (PCA) and hierarchical cluster analysis (HCA)	The first PCA component explained 54.8–58.9% of the variance. HCA grouped *E. crassipes*; mixed vegetation; and other plant targets	Spectral slopes were more effective than indices and bands in distinguishing *E. crassipes*, especially in mixed vegetation zones. Sentinel-2 can be used for species-level classification of aquatic invasive species
Mu et al. (2023)	China, Changguangxi Wetland	ASD Field Spec Pro FR, S185	Classification of *E. crassipes* and other plants	Decision tree	OA, 85%; *E. crassipes* PA, 70%	Lowest classification accuracy for *E. crassipes* due to spectral overlaps with other floating species; *E. crassipes* presence associated with eutrophic, stagnant waters; consistency between ground and UAV spectral data enables monitoring and early assessments

Details include study area, sensor, application, technique, performance, and ecological and remote sensing insights

Multispectral satellite remote sensing has played a central role in shaping current methodologies for monitoring *Eichhornia crassipes*, offering a repeatable and spatially scalable approach to assess plant distribution and dynamics. While not as spectrally dense as hyperspectral sensors, multispectral systems such as Landsat, Sentinel-2, and PlanetScope capture key reflectance regions, particularly NIR, red-edge, and SWIR (Landsat and Sentinel), that are sensitive to vegetation structure, biomass, and moisture content. These features are especially pertinent for *E. crassipes*, whose floating morphology and high chlorophyll content produce distinct spectral signals. Studies (Bayable et al., 2023; Dube et al., 2018) illustrated how Landsat or Sentinel-2 data, even at moderate resolutions, can distinguish phenological stages of *E. crassipes* and map its extent in relation to land cover changes. Sentinel-2’s inclusion of red-edge bands was shown to improve accuracy by nearly 10% compared to Landsat, confirming the advantage of additional strategically designed spectral configurations.

Beyond static detection, multispectral data has proven effective in capturing environmental and temporal dynamics that influence *E. crassipes* proliferation. Ghoussein et al. (2019) applied fractional vegetation cover analysis using Sentinel-2 to differentiate riparian vegetation and floating mats along seasonal growth cycles, revealing spectral slope shifts associated with plant health. Similarly, Worqlul et al. (2020) used PlanetScope imagery to correlate *E. crassipes* coverage with lake level, turbidity, and temperature variations, demonstrating how multispectral satellites can support ecohydrological analyses. These applications emphasize that even with fewer bands, well-timed acquisitions and appropriate indices, such as NDVI or Fractional Vegetation Cover (FVC), can reflect both biological condition and environmental context. This adaptability makes multispectral platforms suitable for both operational surveillance and ecological research in aquatic systems.

Importantly, the simplicity of multispectral indices and the continuity of satellite archives allow researchers to link *E. crassipes* presence with long-term land use, pollution, and hydrological trends. Kleinschroth et al. (2021), for example, used three decades of Landsat NDVI data to show a robust correlation between *E. crassipes* presence and urban wastewater inputs in small reservoirs worldwide. Mukarugwiro et al. (2021) found that manual removal offered only short-term relief, with reinfestation occurring in unmanaged, nutrient-rich waters. As these studies demonstrate, the strength of multispectral satellite data lies not just in its accessibility but in its ability to contextualize *Eichhornia crassipes* within broader environmental processes, enabling a more integrated approach to invasive species monitoring and aquatic ecosystem management.

Sentinel-1 SAR has emerged as a critical tool for monitoring *Eichhornia crassipes* across large aquatic systems due to its ability to acquire high-resolution imagery independent of weather conditions and daylight, addressing key limitations of optical sensing. This capability is especially relevant in tropical regions, where persistent cloud cover can hinder optical surveillance. Studies in both Vembanad Lake, India (Simpson et al., 2022), and the Winam Gulf of Lake Victoria, Kenya (Felix et al., 2023), have demonstrated that SAR can detect dense mats of *E. crassipes* from open water through variations in volumetric backscattering. However, while these papers show promise, they largely focus on regions with expansive and visually dominant infestations. The dependence on visual ground-truthing and assumed spectral homogeneity of *E. crassipes* in these environments may limit the scalability of the methods to more heterogeneous or fragmented ecosystems where species coexist or where mixed aquatic vegetation mats occur.

Despite these limitations, SAR’s spatial and temporal resolution has enabled practical advances in mapping and time-series tracking of *Eichhornia crassipes*. In particular, long-term Sentinel-1 acquisitions spanning several years have facilitated assessments of infestation variability and spread, as seen in the mapping of *E. crassipes* distribution over 5 years in Lake Victoria (Felix et al., 2023) and across seasonal intervals in Indian wetlands (Akbari et al., 2021). These datasets enable broad-scale ecological surveillance, yet they also reveal challenges in discerning *E. crassipes* from spectrally and structurally similar aquatic plants. In Vembanad Lake (Akbari et al., 2021), the authors demonstrated that temporal backscatter variations in the VV channel correspond closely with *E. crassipes* coverage, enabling unsupervised classification of infested areas. Moreover, the utility of SAR remains constrained by its sensitivity to environmental noise such as wind-induced capillary waves, which can interfere with volumetric backscatter and detection thresholds. While polarimetric enhancements and multi-temporal imaging mitigate some of these effects, the reviewed literature underrepresents the implications of such confounding factors, which are crucial for developing operational remote sensing strategies for invasive aquatic plant management. Table 2 summarizes key studies in the application of Sentinel-1 SAR data for monitoring *E. crassipes*.

**Table 2:** Studies applying Sentinel-1 SAR data for *E. crassipes* monitoring

Authors	Study area	Sensor	Application	Techniques	Performance/findings
Simpson et al. (2022)	Indonesia, Batujai reservoir	Sentinel-1	Detection of *E. crassipes*	Change detection algorithms: power difference (PDiff), power ratio (PR), and Hotelling–Lawley trace (HLT)	Change detectors achieved true positive detection rates of 90–98% for high-density infestations. However, in low-density infestations, the detection accuracy dropped to 35–40%. *E. crassipes* hotspots were in low-flow areas and near agricultural barrages and showed expansion during wet periods
Felix et al. (2023)	Kenya, Lake Victoria	Sentinel-1, Sentinel-2	Detection of *E. crassipes*	Optimized power difference (OPDiff) change detection	The OPDiff method achieved a precision of 92.6% and an accuracy of 89.9% compared to Sentinel-2 data. SAR and optical can be integrated for EC monitoring
Belayhun and Mekuriaw (2024)	Ethiopia, Lake Tana	Sentinel-1, Sentinel-2	Distribution modeling of *E. crassipes*	RF, boosted regression tree (BRT), SVM, and ensemble	RF performed better. Area under the curve of 0.93 (dry season) and 0.95 (wet season). Important to consider climatic variables and sentinel bands and indices (B5, B12, VH, ND water index (NDWI), ND adjustment vegetation index (NDAVI)) in EC distribution modeling
Mqingwana et al. (2024)	South Africa, Hartbeespoort Dam	Sentinel-1, Sentinel-2	Classification of *E. crassipes* pre- and post-biological control implementation	RF, SVM, NB (Naïve Bayes), CART	RF achieved the highest accuracy (93.42 to 98.70%) for classification, outperforming SVM, CART, and NB. NB was the least accurate (87.76–92.08%). Biological control with *Megamelus scutellaris* reduced *E. crassipes* coverage. Spectral separability was strongest in NIR and red-edge bands
Aydın (2024)	Türkiye, Orontes River	Sentinel-1, Sentinel-2, Landsat-8, PlanetScope	Detection of *E. crassipes*	Time series: NDVI, NDCI, VV, and VH	Sentinel-1 backscatter trends aligned with Sentinel-2 NDVI peaks, confirming vegetation cover. NDCI peaks preceded NDVI increases, indicating rising chlorophyll-a before hyacinth emergence. Optical and SAR data combined allowed robust, year-round detection despite cloud interference. PlanetScope’s high resolution (3 m) enabled detailed mapping of hyacinth density and spread

Details include study area, sensor, application, technique, performance, and ecological and remote sensing insights

## Algorithms for *E. crassipes* classification: from indices thresholds to deep learning

**Fig. 5:**
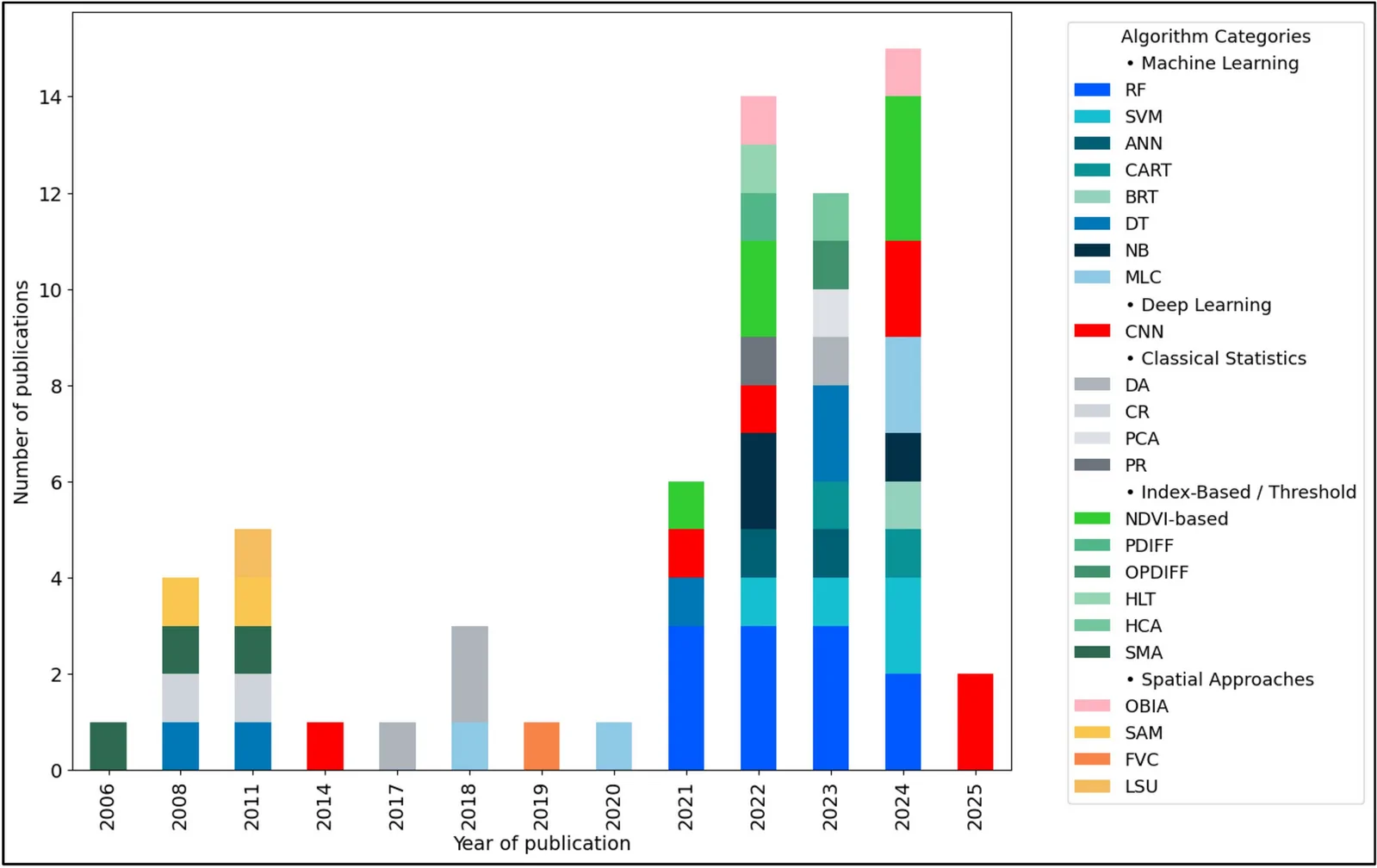
Annual Distribution of Publications by Classification Algorithm for *Eichhornia crassipes* Monitoring (2006–2025). Research has shifted from early index-based, classical statistics toward machine learning approaches such as RF, SVM and CNN. NDVI-based thresholds remain common, reflecting their simplicity and scalability. Notes: MLC, maximum likelihood classification; DA, discriminant analysis; LSU, linear spectral unmixing

The effective classification of *Eichhornia crassipes* in remote sensing imagery depends not only on the quality of the sensor data but also on the analytical methods applied to extract relevant information. Over the past two decades, the field has evolved from relying primarily on simple index-based thresholding approaches, particularly those using the NDVI, to more advanced machine learning (ML) and deep learning (DL) techniques. NDVI-based thresholds remain widely used for initial detection due to their simplicity and ease of interpretation, especially in large-scale or time series applications. However, as spectral, spatial, and temporal data complexity has increased, more adaptive and robust classification algorithms have become essential. As illustrated in Fig. 5, recent years have seen a sharp rise not only in the number of publications applying ML and DL models, such as Random Forest (RF), Support Vector Machines (SVM), and Convolutional Neural Networks (CNN), but also in continued reliance on NDVI and related indices. The growing diversity of algorithmic approaches reflects a shift toward maximizing detection accuracy, minimizing spectral confusion in mixed-vegetation environments, and enabling scalable, operational monitoring of aquatic invasive species like *E. crassipes.*

The NDVI, defined as (NIR − Red)/(NIR + Red), remains a fundamental and widely applied tool for detecting and monitoring *Eichhornia crassipes* in aquatic environments. Its conceptual simplicity, combined with its strong physiological grounding—capturing chlorophyll-related absorption in the red and high reflectance in the near-infrared—has made it especially effective in identifying floating vegetation. NDVI’s compatibility with long-standing Earth observation programs, particularly the Landsat archive dating back to 1984, has enabled the construction of multi-decadal time series for tracking *E. crassipes* dynamics across diverse ecosystems. Gerardo and de Lima (2022) used NDVI thresholds above 0.4 to detect floating vegetation in the Mondego River, while Kleinschroth et al. (2021) adopted a 0.3 threshold to identify *E. crassipes* and similar species in reservoirs worldwide. These threshold-based approaches are effective for large-scale monitoring but face significant limitations in environments where multiple invasive aquatic plants coexist. Species such as *Pistia stratiotes* and *Ludwigia peploides* may exhibit similar spectral responses in the red and near-infrared bands, leading to classification uncertainty in mixed vegetation zones. Moreover, the spatial dynamics of free-floating vegetation, which can shift daily due to wind and hydrological flows, present additional challenges for temporal aggregation methods such as median compositing, employed in Bayable et al. (2023). These techniques may obscure short-term fluctuations that are critical for accurately detecting the extent and movement of *E. crassipes* mats.

The study by Aydın (2024) reinforces the utility of NDVI for detecting and tracking *Eichhornia crassipes*, demonstrating its effectiveness through multi-year time series from Sentinel-2, Landsat-8, and PlanetScope imagery. Notably, the integration of Sentinel-2’s red-edge bands enabled the application of the Normalized Difference Chlorophyll Index (NDCI), which consistently preceded NDVI increases and served as an early indicator of chlorophyll-a enrichment before *E. crassipes* increased propagation. A similar behavior was noted by Mucheye et al. (2022) in Lake Tana, Ethiopia, using Sen2Cor atmospheric processor, where peaks of chlorophyll-a and total suspended matter occurred 1 month before the *E. crassipes* coverage and biomass peaks. Complementary use of Sentinel-1 SAR backscatter data further enhanced temporal continuity under cloud cover, with VV and VH polarization trends aligning with optical vegetation indices. While the combined use of NDVI, NDCI, and SAR proved robust in capturing seasonal dynamics and phenological transitions of *E. crassipes*, future studies should consider incorporating indices designed explicitly for aquatic backgrounds, such as the Normalized Difference Aquatic Vegetation Index (NDAVI) and the Water Adjusted Vegetation Index (WAVI), which replace or adjust the visible bands of NDVI and Soil-Adjusted Vegetation Index (SAVI) to suppress the water signal and improve separability between aquatic and terrestrial vegetation. Complementary surface-floating indices, such as the Floating Algal Index (FAI), may further isolate emergent mats from open water under variable turbidity. Although still underutilized in *E. crassipes* monitoring, these aquatic-specific metrics offer a more physically grounded spectral framework than land-oriented indices for operational detection in optically complex waters and mixed vegetation zones.

Beyond presence detection, NDVI has also proven useful for estimating biomass and quantifying vegetation cover over time. In Lake Tana, Ethiopia, Mucheye et al. (2022) demonstrated that NDVI values ranging from 0.6 to 0.95 tracked seasonal biomass fluctuations, reflecting environmental changes in chlorophyll concentration and turbidity. Similarly, Pádua et al. (2022b) showed that the median NDVI of a reservoir is strongly correlated with the percentage cover of *E. crassipes* (*R*^2^ = 0.91), supporting its application as a simple and effective proxy for mapping infestation extent. These findings underscore NDVI’s value not only for detection but also for ecological assessment, though it needs more advanced indices and multi-sensor approaches to improve robustness in heterogeneous and temporally dynamic aquatic systems.

Machine learning algorithms have been widely adopted for classifying *Eichhornia crassipes* in remote sensing imagery, particularly with the increasing availability of high-resolution multispectral data from platforms such as Sentinel-2 and Landsat 8. Among the most frequently used methods are Random Forest (RF), Support Vector Machines (SVM), Classification and Regression Trees (CART), *k*-nearest neighbors (KNN), Naive Bayes (NB), and Artificial Neural Networks (ANN). These algorithms have demonstrated high classification accuracies, often exceeding 85%, although performance varies depending on the choice of input features, spatial resolution, and environmental context. For instance, Bayable et al. (2023) and Radampola et al. (2023) reported strong results using RF across wet and dry seasons, highlighting the algorithm’s flexibility in capturing phenological changes. Pádua et al. (2022a) tested multiple models using Sentinel-2 imagery and observed comparable performance between RF and ANN, with classification accuracies around 86–90%, while KNN and NB yielded slightly lower results. Belayhun and Mekuriaw (2024) explored an ensemble of RF, SVM, and boosted regression trees (BRT), confirming the relevance of multi-model approaches for improving reliability.

Vipra et al. (2024) further showed that Sentinel-2 Level-2A imagery can support ML-based invasive aquatic weed detection when NDVI is combined with optimum index factor (OIF) based band selection, which identified the Green, Red, and NIR bands as the most informative and least redundant composite. Using this OIF–NDVI feature set, XGBoost slightly outperformed ANN for binary weed detection, although the broad “weed” class and absence of independent spatial validation limit its interpretation as species-specific *E. crassipes* mapping. A recent RF application partitioned the *E. crassipes* life cycle into four field-defined stages (early, mid, high, and decaying), each carrying a distinct spectral signature, as chlorophyll content rises through the growing phases and falls during senescence, shifting near-infrared reflectance and NDVI accordingly. Mapping stage rather than mere presence supports early warning, allowing intervention before the high and flowering stages, when control becomes most difficult (Pirbasti et al., 2024).

Collectively, these studies suggest that while no single ML model is universally superior, RF remains one of the most frequently applied due to its interpretability, ease of implementation, and ability to handle high-dimensional spectral and index-based inputs. Nonetheless, algorithm selection remains context-dependent, and performance is often influenced by seasonal variation, the presence of spectrally similar vegetation, and the availability of high-quality training data.

Reported accuracies across these studies warrant cautious interpretation, as headline figures often overstate operational performance on *E. crassipes* specifically. Overall accuracy is dominated by the open-water and background classes that constitute most of any aquatic scene, so values above 95% (Bayable et al., 2023; Godana et al., 2022; Radampola et al., 2023) reveal little about the target class in isolation. Class-specific metrics expose this gap: Godana et al. (2022) reported overall accuracy near 99% yet a user’s accuracy of 67% for *E. crassipes* in 1 year, indicating that a third of detections were false positives, while Mu et al. (2023) recorded 85% overall accuracy but only 70% producer’s accuracy for *E. crassipes*, the lowest among eight species owing to confusion with co-occurring floating plants. Validation design compounds the problem. Where reference samples are drawn from the same imagery being classified rather than from independent field data, the dominant error escapes the assessment entirely; Radampola et al. (2023) reported F1 scores near 98% yet explicitly noted that the omission of sub-pixel patches could not be captured by their image-derived validation. SAR detection rates are similarly conditional: Simpson et al. (2022) achieved true-positive rates of 90–98% over dense, manually delineated infestations, but the same detectors fell to 35–40% in low-density patches, the regime most relevant to early warning. Robust performance assessment therefore requires reporting producer’s and user’s accuracy, F1, or IoU alongside overall accuracy, and validation on spatially independent samples, ideally with better spatial resolution—practices that remain inconsistent across the reviewed corpus and that constrain direct cross-study comparison.

In this context, object-based image analysis (OBIA) has emerged as a highly accurate classification technique for remote sensing data, particularly for aquatic invasive species. By segmenting imagery into meaningful objects based on shape, texture, and spectral properties, OBIA enables more detailed and context-aware analysis compared to traditional pixel-based methods. For instance, Godana et al. (2022) applied OBIA to Sentinel-2 imagery in Lake Dambal, achieving detection accuracies of water and *E. crassipes* exceeding 99%. Similarly, Mqingwana et al. (2024) implemented OBIA combined with RF and SVM classifiers in the Hartbeespoort Dam, attaining accuracies ranging from 93.42 to 98.70%, slightly higher than pixel-based methods. Their approach minimized misclassification errors caused by mixed pixels and improved the monitoring of floating plant mats in heterogeneous aquatic environments.

These methodological advances reflect the growing sophistication in the use of multispectral imagery for monitoring *Eichhornia crassipes*, integrating both spectral indices and classification algorithms across various environmental settings. Table 3 summarizes key studies that applied multispectral data—whether from satellite, UAV, or hybrid sources—highlighting the sensors, techniques, classification performance, and main conclusions drawn from each application.

**Table 3:** Studies applying multispectral sensors for *E. crassipes* monitoring

Authors	Study area	Sensor	Application	Techniques	Performance	Findings
Ghoussein et al. (2019)	Al Kabir River, Syria and Lebanon	Sentinel-2, Google Earth	Detection of *E. crassipes*	Fractional vegetation cover (FVC)-SAVI	In a narrow river, it showed high accuracy (*R* = 0.97, RMSE*P* = 0.059 ha) when validated against Google Earth	Able to distinguish *E. crassipes* from riparian vegetation due to differences in their vegetative cycles and development periods. The use of SAVI instead of NDVI reduced sensitivity to water turbidity and riparian soil background, improving FVC estimates in aquatic environments
Kleinschroth et al. (2021)	20 reservoirs worldwide	Landsat 5, 7, 8	Classification of *E. crassipes* and other classes; correlation with environmental drivers	NDVI detection thresholds of ≥ 0.3; linear correlations analysis	Peak floating vegetation cover showed a strong positive correlation with urban land cover (*R*^2^ = 0.43). Smaller reservoirs (< 5 km^2^) were more affected by floating vegetation, reaching over 80% vegetation cover (*R*^2^ = 0.61)	*E. crassipes* proliferation was driven by nutrient pollution from urban wastewater, especially in small, low-flow reservoirs. Reservoir type influenced growth, with smaller reservoirs more prone to nutrient-driven expansion. Floating vegetation indicated eutrophication and temporarily buffered nutrients while promoting invasive growth
Mucheye et al. (2022)	Ethiopia, Lake Tana	Sentinel-2	Detection of *E. crassipes*; Biomass estimation; Correlation with environmental drivers	Reservoir-mean NDVI; NDVI biomass estimation from 0.6 to 0.95; linear correlation analysis	Chlorophyll-a concentration (*r* = 0.53) and TSM (*r* = 0.51) were moderately correlated with *E. crassipes* coverage, indicating nutrient availability as a growth driver	NDVI values (0.60–0.95) enable biomass estimation; reservoir mean NDVI time series clearly reflected seasonal phenology, with growth starting in the rainy season (July–August), peaking in October, and declining in dry months; despite reported removal efforts, post-removal NDVI data indicated rapid regrowth
Godana et al. (2022)	Ethiopia, Lake Dambal	Sentinel-2	Classification of *E. crassipes* and other classes	Object-based image analysis; linear correlation analysis	OA ranged from 91.7 to 99.3%, with *E. crassipes* PA between 82.4 and 100%. *E. crassipes* coverage positively correlated with lake surface area (*r* = 0.41)	Seasonal analysis showed that *E. crassipes* cover increased during wet seasons, driven by nutrient inflows and seasonal water level fluctuations
Janssens et al. (2022)	Vietnam, Saigon River	Sentinel-2, Worldview-2	Detection of *E. crassipes*; correlation with environmental drivers	Naïve Bayes (NB); linear correlation analysis	The NB detector achieved 91% accuracy. *E. crassipes* coverage correlated negatively with rainfall (*r* = −0.58) and humidity (*r* = −0.61), and positively with water organic material (*r* = 0.78)	In this river environment, *E. crassipes* coverage peaked in the dry season under low-flow conditions, while wet periods disrupted it. Positive links with organic material highlighted its nutrient role
Pádua et al. (2022a)	Portugal, Mondego River basin	MicaSense RedEdge-MX, Sentinel-2	Detection *E. crassipes*	RF, ANN, SVM, NB, k-NN	RF had the highest accuracy (94% UAV, 90% Sentinel-2). All classifiers achieved OA > 83%	Sentinel-2 enabled reliable large-scale monitoring despite its lower spatial resolution. UAV imagery detected small hyacinth patches missed by satellite data. Temporal integration revealed hyacinth spread reduction near containment barriers
Pádua et al. (2022b)	Portugal, Mondego River basin	MicaSense RedEdge-MX, Sentinel-2	Detection of *E. crassipes*;	RF and NDVI detection thresholds of ≥ 0.75; linear correlation analysis	NDVI thresholding (97.8%) outperformed RF (93.3%). Sentinel-2 and UAV NDVI showed strong agreement (*R*^2^ = 0.89), and *E. crassipes* cover strongly correlated with the mean NDVI of water channels (*R*^2^ = 0.91)	Mean NDVI proved a reliable indicator of aquatic plant cover. UAV data detected fragmented hyacinth patches, while Sentinel-2 supported large-scale monitoring. Retention structures in 2021 stabilized growth and reduced spread
Radampola et al. (2023)	Sri Lanka, North Bolgoda Lake	Sentinel-2	Detection of *E. crassipes*	Pixel-based RF	OA exceeded 98% for both wet (98.7%) and dry (98.2%) seasons. UA was 96.38–97.7%, and F1 scores were 98.2–98.9%	NDVI, ND built-up index (NDBI), enhanced vegetation index (EVI), B8 and Red edge1/Red (RR1) were the most important features
Bayable et al. (2023)	Ethiopia, Lake Tana	Landsat-8, Sentinel-2	Classification of *E. crassipes* and other classes	RF, SVM and CART	For Sentinel-2 images, classifiers achieved > 95% OA. F1 *E. crassipes* 99% (autumn) to 94% (winter). For Landsat 8, all methods achieved > 90% OA	Sentinel-2’s red-edge bands improved detection accuracy over Landsat 8, with RF performing slightly better

Deep learning (DL) techniques have recently emerged as powerful tools for segmenting and classifying *Eichhornia crassipes* in high-resolution remote sensing imagery, offering advantages in automation, spatial generalization, and pixel-level precision. In particular, convolutional neural networks (CNNs) such as U-Net, PSPNet, DeepLabV3+, and SegNet have been adapted to process UAV-based multispectral and RGB imagery. Fu et al. (2021) demonstrated that a Random Forest Decision Tree (RF-DT) outperformed SegNet in karst wetland classification, but also revealed the importance of multi-source features, including texture, shape, and DSM data, in enhancing model performance for floating vegetation detection. Meanwhile, Li et al. (2022) showed that CNN architectures—especially PSPNet and DeepLabV3+—achieved higher Intersection over Union (IoU) scores (up to 81.93%) when integrated with DSM and texture layers. Their findings emphasized the strength of one-class classification (OCC) strategies, which yielded 3.24 to 10.97% accuracy improvements over multi-class approaches. These studies collectively suggest that while shallow models may remain competitive under well-structured feature engineering, deep CNNs offer superior robustness in complex wetland conditions, particularly when trained with multidimensional datasets.

Recent advances have also highlighted the value of tailored CNN architectures that explicitly integrate spatial and spectral information. Herrera Ollachica et al. (2025) developed a custom-built multispectral UAV platform to test a U-Net-based segmentation pipeline with five bands, including NIR at 720 and 850 nm. They reported a mean IoU of 97%, demonstrating that U-Net could outperform traditional ML algorithms when applied to pixel-level semantic segmentation of *E. crassipes*. Their study also addressed the role of NDVI-derived masks as high-quality references to enhance model supervision, offering a way to overcome the limitations of manual annotations. Complementarily, Rodriguez-Garlito et al. (2023) explored CNN1D and CNN2D models trained on Sentinel-2 data to detect invasive aquatic species over multi-temporal scales. Their CNN2D model achieved 95.9% accuracy and an F1 score of 94.6% in the Mérida case study, underlining the impact of spatial–spectral fusion. Notably, CNN2D consistently outperformed CNN1D, especially in detecting fragmented plant patches, confirming that spatial context is essential for distinguishing *E. crassipes* from co-occurring vegetation. These results position CNNs not only as effective classifiers but also as scalable, adaptive tools suitable for continuous environmental monitoring using both UAV and satellite datasets.

Atmospheric correction remains a fundamental step in remote sensing of aquatic invasive plants because it affects the spectral values used in vegetation indices, classification thresholds, model calibration, and cross-sensor comparisons. Although this issue has been less explicitly discussed in recent studies due to the increasing availability of analysis-ready or atmospherically corrected products, it is still critical in optically complex aquatic environments, where turbidity, aerosols, adjacency effects, and sun glint can alter the contrast between floating vegetation, open water, sediments, and phytoplankton, as shown in highly turbid waters using Rayleigh-corrected reflectance and ACOLITE processing (Dogliotti et al., 2018). For time-series monitoring of *E. crassipes*, bottom-of-atmosphere Sentinel-2 products generated with MAJA/MACCS or Sen2Cor have been used to improve temporal consistency and reduce confusion with riparian vegetation and water-quality effects (Ghoussein et al., 2019). Similarly, when *E. crassipes* dynamics are linked to water-quality variables, dedicated processors such as Sen2Cor and C2RCC are particularly important because differences between TOA and corrected reflectance products can affect NDVI-based cover estimates as well as chlorophyll-a and total suspended matter retrievals (Mucheye et al., 2022).

## Multi-platform remote sensing of *E. crassipes*

In recent years, the remote sensing community has increasingly adopted integrative approaches that combine multiple sensing platforms, sensor types, and analytical techniques to enhance the detection and monitoring of *Eichhornia crassipes*. These strategies are particularly valuable in aquatic environments with small vegetation patches, where environmental variability and spectral overlap among co-occurring vegetation challenge single-sensor detection methods.

**Fig. 6:**
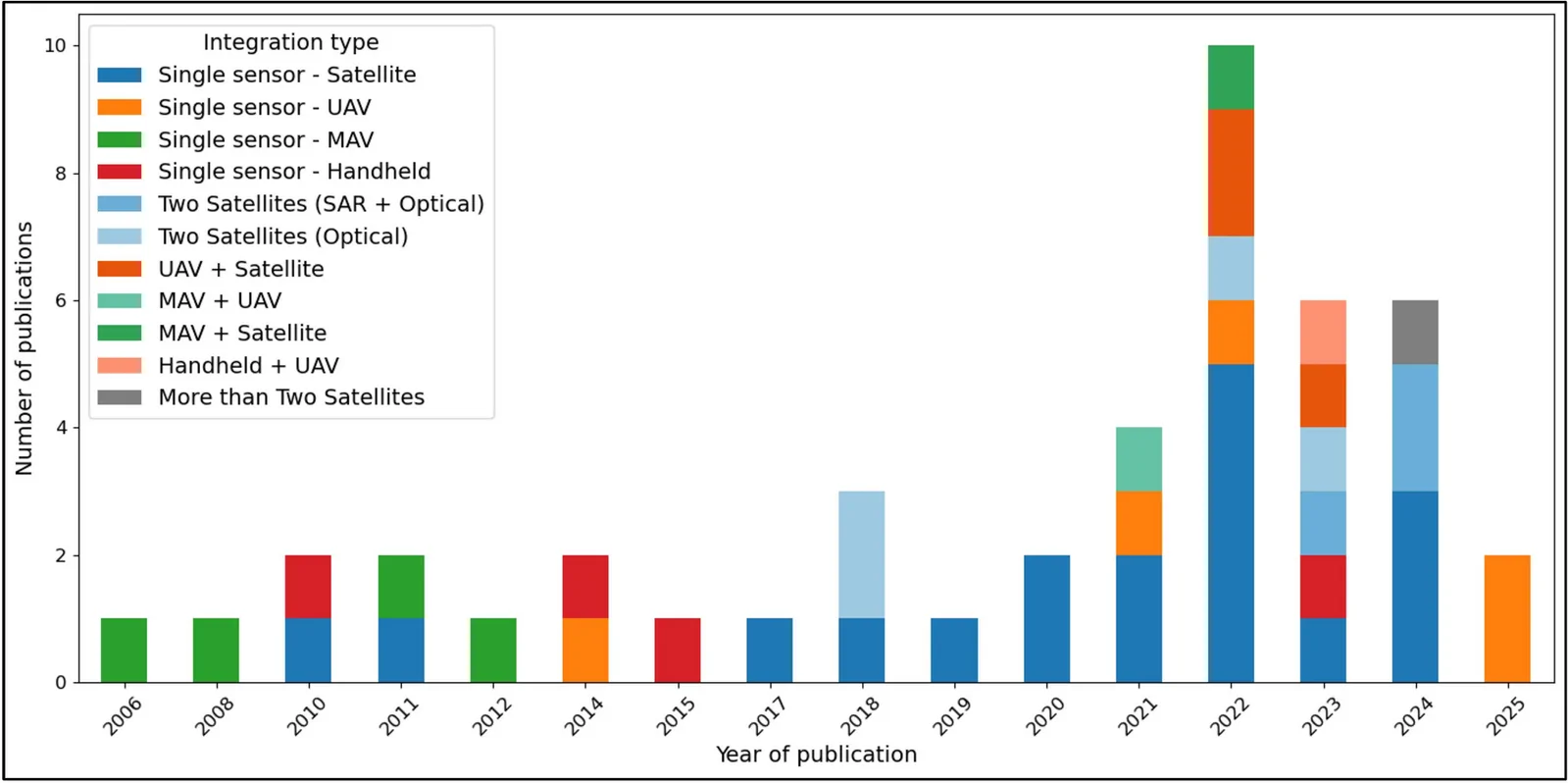
Evolution of single- and multi-sensor platform use in remote sensing studies on *Eichhornia crassipes.* Early studies relied mainly on single-sensor approaches, especially satellites, handheld devices, and MAVs. Since 2021, multi-platform integrations, most notably UAV + satellite and satellite constellations (SAR + optical), have increased

Figure 6 illustrates the annual distribution of publications categorized by platform and sensor integration types from 2006 to 2025. While early research was dominated by single-sensor applications—primarily satellites, handheld devices, and MAVs—a noticeable shift began around 2018. From 2021 onward, studies increasingly explored multi-platform combinations such as UAV + satellite, MAV + UAV, and satellite constellations (SAR + optical), indicating a growing preference for complementary data integration. Considering the papers analyzed here, 37% were performed with two platforms.

Recent studies further illustrate how multi-platform approaches are moving beyond accuracy-oriented classification toward ecological assessment and management support. In Honghu Lake, the integration of Sentinel-1 SAR and Sentinel-2 MSI with UAV-supported field interpretation and PlanetScope imagery enabled the mapping of aquatic vegetation, including wild water hyacinth, from 2019 to 2024, revealing major shifts in open water, sediment exposure, and floating macrophyte dominance as evidence for lake-specific conservation planning (Keyi et al., 2025). A complementary nested satellite–UAV strategy has also been demonstrated in riparian habitats, where Sentinel-2 data processed in Google Earth Engine were used to delineate priority habitat clusters at the riverscape scale, followed by UAV-based OBIA classification for fine-scale assessment of aquatic vegetation, water hyacinth, submerged vegetation, riparian vegetation, grasslands, and agricultural conversion (Tripathi et al., 2024). Although not focused exclusively on *E. crassipes*, this approach reinforces the operational value of multi-platform monitoring by showing how satellite imagery can guide UAV deployment, reduce field effort, and generate spatially explicit evidence for conservation planning and early threat detection.

Studies have demonstrated the advantages of combining high-resolution platforms like UAVs and MAVs with multispectral satellite data to improve *Eichhornia crassipes* detection. Bolch et al. (2021) reported that Nano-Hyperspec UAV data achieved higher overall accuracy (94%) than the HyMap MAV (85%) in the Sacramento–San Joaquin River Delta, offering finer spatial detail for EC mapping. Similarly, Ade et al. (2022) integrated HyMap with Sentinel-2 data and found that while the hyperspectral MAV outperformed Sentinel-2 alone in classification accuracy, the satellite imagery was useful for filling intra-annual temporal gaps. These studies underscore the benefit of integrating fine-resolution aerial and ground sensors with broad-coverage satellite imagery for more comprehensive monitoring strategies.

Further supporting this integration approach, Janssens et al. (2022) validated Sentinel-2 classifications of *Eichhornia crassipes* in the Saigon River using high-resolution WorldView-2 imagery, achieving 91% accuracy and effectively capturing seasonal dynamics. Likewise, Ghoussein et al. (2019) used Google Earth imagery as a reference to validate Sentinel-2-based FVC estimates of *E. crassipes* coverage, reporting a strong correlation (*R* = 0.97) in complex river systems. These studies reinforce the value of combining moderate-resolution satellite imagery with high-resolution reference data to enhance classification accuracy and detect temporal vegetation changes in heterogeneous aquatic environments.

The integrative use of UAV and satellite data has been methodologically refined in the dual studies by Pádua et al. (2022a, 2022b), who explored both the technical complementarities and comparative accuracies of these platforms for monitoring *Eichhornia crassipes*. In the first study, UAV data acquired with a MicaSense RedEdge-MX sensor were directly compared with Sentinel-2 MSI imagery over synchronized timeframes, allowing a rigorous evaluation of spatial detail versus temporal frequency (Pádua et al., 2022a). The authors implemented multiple classifiers, including RF and SVM, across both datasets, revealing that despite the UAV’s superior spatial resolution enabling more precise delineation of small patches, Sentinel-2 remained highly effective and reliable when atmospheric conditions permitted, especially when resampled or paired with vegetation indices. The second study advanced this integration by calibrating NDVI thresholds using UAV data to automate long-term Sentinel-2 time series analysis (Pádua et al., 2022b).

In addition to that, Mouta et al. (2023) used 5 cm UAV-derived orthomosaics to delineate training areas for Sentinel-2 classification. By linking fine-scale UAV observations with satellite imagery, the authors improved spectral signature refinement and supported satellite-based binarization models, reducing dependence on labor-intensive ground-truthing. This same logic of using finer-resolution imagery to calibrate medium-resolution satellite products was adopted in combination with classified 0.25-m aerial orthophotos over the Guadiana River, Spain, and resampled by majority voting to the 10-m Sentinel-2 grid. This produced synthetic ground truth for evaluating Sentinel-2 maps of *E. crassipes* and *Nymphaea mexicana* (Rodríguez-Garlito et al., 2022). Together, these studies show that UAV and aerial reference imagery can bridge the scale gap between field observations and Sentinel-2 time series, improving calibration, validation, and operational monitoring of invasive aquatic plants.

## Linking remote sensing and environmental data

Understanding the dynamics of *Eichhornia crassipes* across aquatic ecosystems requires linking environmental variables with physiological responses detectable by remote sensing. Observation platforms have become key tools for capturing changes in *E. crassipes* coverage and health, providing insights into both natural drivers, such as hydrology, climate, and nutrient availability, and anthropogenic interventions, including mechanical removal efforts and herbicide and pollutant exposure. Correlating vegetation indices with environmental data and stress signals offers a scalable and objective basis for managing invasive aquatic vegetation.

Hydrological conditions, particularly lake levels, consistently emerge as a key determinant of *E. crassipes* proliferation. Dersseh et al. (2020) found a significant linear correlation (*R* = 0.69; *p* < 0.01) between lake levels and NDVI-derived coverage, suggesting that higher water levels expand suitable habitats for *E. crassipes*. Similarly, Godana et al. (2022) observed a positive correlation (*r* = 0.41) between *E. crassipes* coverage and lake surface area, influenced by fluctuating water levels. These findings align with the work of Worqlul et al. (2020), who reported a strong positive relationship between *E. crassipes* coverage and lake levels (*R*^2^ = 0.75) as well as turbidity with a 2-month lag (*R*^2^ = 0.74). This indicates that increased sediment inflow during wet seasons creates nutrient-rich conditions favorable for *E. crassipes* expansion.

Nutrient availability and climatic factors also significantly influence *E. crassipes* proliferation. Mucheye et al. (2022) found a moderate positive correlation (Pearson coefficient = 0.53; *p* = 0.05) between NDVI-based *E. crassipes* coverage and chlorophyll-a (Chl-a) and total suspended matter (TSM), highlighting the role of nutrient inflows in driving biomass accumulation. Similarly, at the Saigon River, Vietnam, Janssens et al. (2022) observed a strong positive correlation (*r* = 0.78; *p* = 0.01) between *E. crassipes* coverage and organic material concentrations, emphasizing the importance of eutrophication in sustaining dense infestations. However, rainfall and humidity negatively correlated with *E. crassipes* coverage (*r* = −0.58 and *r* = −0.61, respectively), suggesting that drier conditions, which limit water flow and enhance nutrient concentration, favor *E. crassipes* dominance.

In worldwide reservoirs study, Kleinschroth et al. (2021) demonstrated that urban and agricultural land cover positively correlated with floating vegetation expansion (*R*^2^ = 0.43; *p* < 0.001), with smaller reservoirs (< 5 km^2^) being particularly vulnerable to nutrient-driven growth (*R*^2^ = 0.61; *p* = 0.002). These correlations highlight the intricate interplay of hydrology, nutrient dynamics, and climatic conditions in driving the spatial distribution of *E. crassipes*, underscoring the need for integrated monitoring approaches to manage its spread effectively.

Beyond explaining environmental drivers of *E. crassipes* proliferation, remote sensing outputs can also be translated into resource-oriented management indicators. In Sri Lanka, high-resolution Planet imagery was used to delineate water hyacinth across 28 water bodies and estimate its biomass and biochar potential (Ghosh et al., 2024). This approach expands the role of remote sensing from monitoring infestation extent to supporting circular-economy strategies, where mapped biomass can inform removal priorities, estimate recoverable plant material, and evaluate opportunities for biochar, composting, bioremediation, or bioenergy production.

To evaluate the physiological response of *E. crassipes* to chemical stressors, including herbicides and pollutants, Newete et al. (2014) conducted a controlled experiment combining biocontrol agents (*Neochetina* spp.) with a range of heavy metal treatments (e.g., Cu, Hg, Zn) and observed that hyperspectral indices such as mNDVI705 and red-edge position (REP) were effective in detecting chlorophyll degradation and stress signatures in *E. crassipes*, even revealing how certain metals reduced weevil feeding activity. Robles et al. (2015) explored the use of NDVI derived from simulated Landsat-5 TM imagery to estimate *E. crassipes* biomass and suggested that combining spectral indices with morphometric parameters (e.g., LAI and leaf length) enhances detection of stress caused by herbicide injury, though NDVI alone was limited in dense canopies. More recently, Riner et al. (2025) used UAS-based RGB imaging to evaluate the effectiveness of six herbicide treatments in mesocosms and found that specific RGB vegetation indices (e.g., carotenoid reflectance index (CRI), visible atmospherically resistant index (VARI)) were strongly correlated with herbicide injury, providing rapid, scalable alternatives to subjective field ratings. Collectively, these three studies show that physiological status and stress responses in *E. crassipes*, whether from pollutants or chemical control agents, can be reliably monitored through spectral traits, offering a pathway for more responsive and ecologically informed management strategies.

## Decision-support frameworks for monitoring and managing *Eichhornia crassipes*

**Fig. 7:**
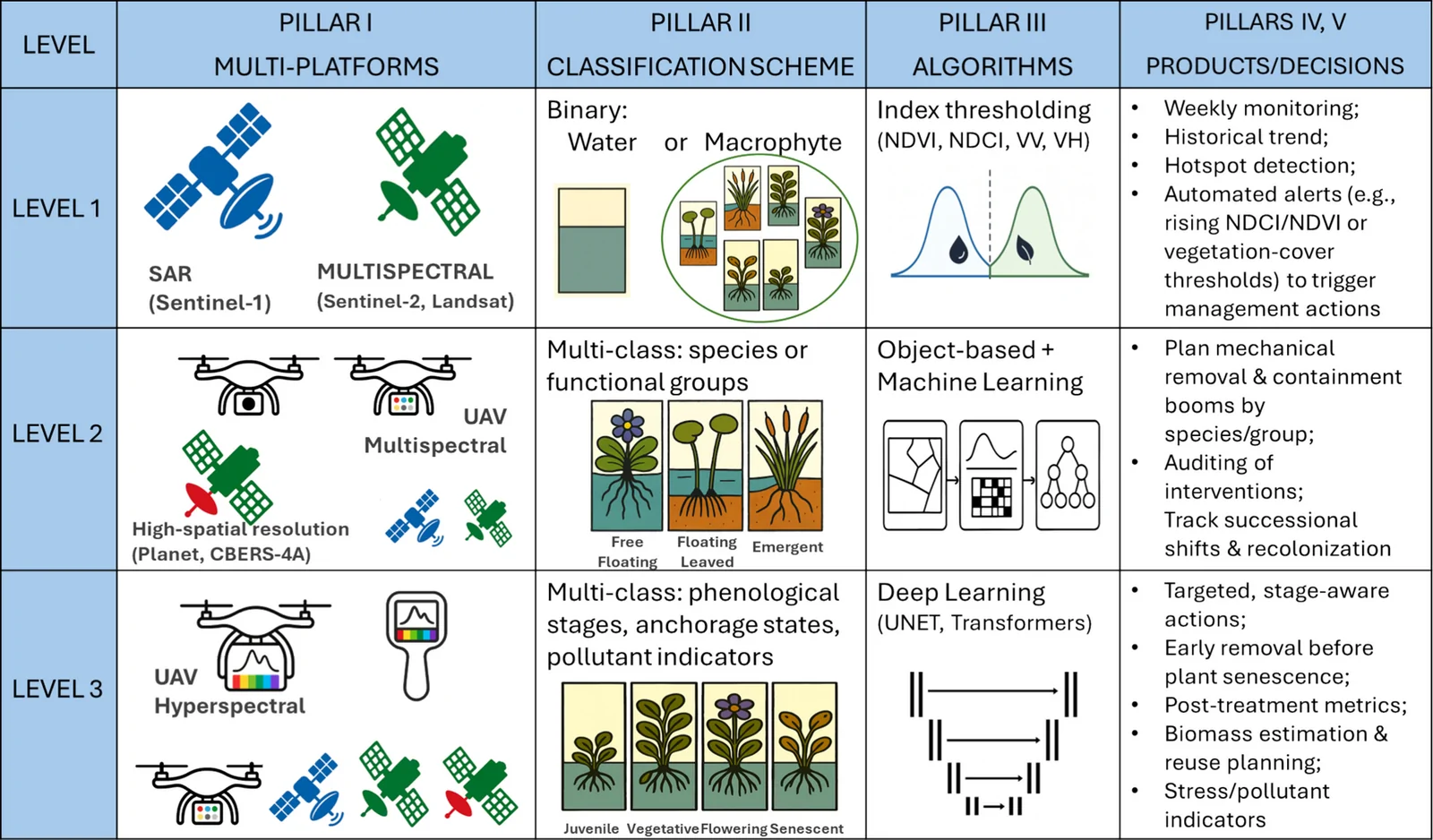
Conceptual decision-support framework for monitoring and managing *Eichhornia crassipes*. The framework is organized around five strategic pillars, spanning multi-platform data fusion, ecologically interpretable classification schemes, scalable analytical approaches, early-warning indicators, and feedback loops linking monitoring to management actions. Three hierarchical levels illustrate increasing analytical complexity and decision relevance, from satellite-based detection to UAV multispectral and hyperspectral analyses for species identification, phenological stage assessment, anchorage state discrimination, and pollutant-related stress indicators

To address the complex ecological, hydrological, and socioeconomic challenges posed by *Eichhornia crassipes*, remote sensing must evolve from simple detection tools into integrated decision-support frameworks for monitoring and management. These systems should not only describe plant distribution but also anticipate change, support the prioritization of control efforts, guide intervention strategies, and evaluate management effectiveness. Figure 7 presents a conceptual decision-support framework built on five strategic pillars, each supporting different stages of monitoring and adaptive management: (i) multi-platform, multi-sensor fusion for consistent and flexible coverage; (ii) a taxonomic, anchorage, and phenological labeling scheme to link remote observations to control actions; (iii) a scalable analytical ladder aligned with scene complexity and intervention risk; (iv) early-warning capabilities based on pre-emergence physiological indicators; and (v) the integration of management actions into the monitoring loop to assess effectiveness and inform future decisions.

### Pillar I—a multi-scale, multi-sensor baseline for operational monitoring

The foundation of any remote sensing framework must be a scalable, multi-platform infrastructure that ensures spatial, temporal, and spectral continuity. At the core lies a fusion of satellite-based multispectral imagery, particularly sensors like Sentinel-2 and Landsat 8, with SAR data, such as from Sentinel-1, which provides robust, weather-independent monitoring. These sources form the backbone for regular, basin-wide assessments. To enhance spatial detail and resolve fine-scale infestations, on-demand UAV imagery equipped with multispectral or hyperspectral sensors should be acquired over priority areas. Proximal and UAV-based data play a critical role in refining class separability and detecting stress conditions not visible in broader-band satellite data.

Practically, this baseline requires the generation of a high-frequency (e.g., weekly) time-series cube that integrates optical and SAR observations. Higher-revisit commercial constellations (e.g., PlanetScope) may be incorporated to improve temporal resolution in cloud-prone or rapidly changing areas. UAV campaigns should be scheduled within the same hour or day window of satellite overpasses to maximize temporal alignment, especially when tracking highly mobile floating mats. Calibration steps must include radiometric correction, geometric normalization, and rigorous co-registration to ensure that UAV-derived labels, thresholds, and class boundaries are reliably transferable to satellite imagery. This tiered approach harmonizes data fidelity with coverage needs and operational scalability.

### Pillar II—encoding species identity, anchorage state, and phenological stage

Remote sensing products must be ecologically interpretable and directly actionable. Therefore, classification schemes should move beyond binary presence/absence to include explicit semantic labels: species identity (e.g., *E. crassipes*, *Pistia stratiotes*, *Typha domingensis*), anchorage state (free-floating, semi-anchored, emergent), and phenological stage (vegetative, flowering, senescent). These labels enable the reconstruction of invasion trajectories, from initial floating mats to rooted stands and competitive dominance over native vegetation. This structured scheme has direct implications for management. Knowing species identity and anchorage type allows the selection of appropriate mechanical removal technologies (e.g., harvesters, booms, netting) and informs regulatory decisions about chemical treatments or biological control agents. Phenological data further contributes by indicating biomass accumulation, nutrient loading, and potential physiological stress induced by environmental contaminants, detectable through specific spectral alterations, thereby supporting timely interventions such as harvesting prior to senescence, minimizing nutrient leaching into the water column, and reducing the risk of pollutant remobilization.

Moreover, embedding this scheme in both training datasets and final map products enhances the interpretability of outputs and improves transparency in management audits. When coupled with biomass estimation models, these maps can also provide logistics-relevant parameters such as tonnage, nutrient load, and removal cost. Biomass metrics enable downstream decision-making about plant reuse (e.g., bioenergy, compost, fiber production), ecological impact evaluation, and post-treatment monitoring, aligning remote sensing products with broader ecosystem services and circular economy principles.

### Pillar III—from indicators to early-warning and forecasting systems

The transition from reactive monitoring to proactive ecosystem management hinges on early-warning capabilities. Multitemporal analyses of aquatic plant dynamics consistently reveal that physiological indicators, such as chlorophyll-a concentration captured via the NDCI, exhibit detectable increases prior to visible canopy development measured by structural indices like NDVI. This lead time, ranging from several days to weeks, offers a valuable opportunity to identify and address emerging infestations before exponential growth or environmental impact.

Integrating water remote sensing indices and in situ measurements into routine monitoring pipelines enables early detection of nutrient enrichment and algal precursors to plant proliferation. Time-series modeling of these indices can be combined with environmental covariates (e.g., rainfall, temperature, water level) to construct predictive models or risk maps. Machine learning methods or mechanistic models can be calibrated to forecast outbreak probability, growth rates, or spatial expansion zones.

Operationally, such forecasts can trigger alerts, prioritize UAV reconnaissance flights, or prompt preventive removal or containment actions. When embedded into an adaptive management framework, early-warning indices transform remote sensing from a descriptive system into a predictive and decision-support infrastructure, enhancing response speed, reducing treatment costs, and minimizing ecological disruption.

### Pillar IV—a scalable analytical ladder for decision-oriented classification

To match algorithmic complexity with ecological variability and management urgency, we propose a three-tiered analytical ladder in which each stage responds to increasing demands for precision, interpretability, and decision relevance. At the first level, aquatic vegetation can be detected through index thresholding using metrics such as NDVI, NDCI, and MNDWI, combined with SAR backscatter variables such as VV and VH in daily to weekly optical–SAR time series. This approach is particularly useful for rapid triage, hotspot identification, and broad-scale tracking of expansion, although it remains limited in species-level and phenological discrimination. A second level incorporates object-based image analysis and classical machine learning models, using spectral, textural, and contextual information to distinguish *E. crassipes* from other aquatic vegetation. At this stage, outputs become more directly relevant to management, as they can support decisions related to intervention timing, the selection of mechanical removal methods, and logistical planning. The third level is designed for ecologically complex settings, such as mixed stands or environments with rapid phenological transitions. Here, deep learning models such as U-Net and DeepLabV3 +, trained on fused multimodal datasets including multispectral, UAV, SAR, and hyperspectral imagery, can simultaneously classify species identity and phenological stage. This level of detail supports more targeted actions, such as prioritizing the removal of senescent biomass to reduce nutrient recycling or containing early-stage infestations before further spread.

### Pillar V—closing the loop with management actions and feedback

Ultimately, the value of remote sensing lies in its capacity not only to detect and describe infestations, but also to inform, guide, and validate ecosystem management. For this reason, remote sensing outputs should be integrated throughout the full management cycle, before, during, and after interventions. Classification products can feed directly into treatment protocols by linking species identity, infestation structure, and phenological stage to appropriate removal technologies and environmental constraints. In practice, the detection of dense *E. crassipes* mats in a senescent phase may support decisions related to biomass recovery and nutrient reuse, whereas the identification of localized pioneer patches may justify rapid containment measures such as netting or biological control. Embedding monitoring outputs within this feedback loop strengthens the adaptive dimension of management, allowing interventions to be continuously reassessed in light of ecological responses and operational outcomes.

## Limitations of the study

Despite the comprehensive synthesis presented, this review is subject to limitations related to both the selection criteria and the analytical approach adopted. The inclusion of studies was restricted to peer-reviewed publications with accessible DOIs and written in English, which, while ensuring traceability and reproducibility, may have introduced selection bias by excluding relevant regional studies, technical reports, and operationally oriented gray literature commonly applied in regions impacted by *Eichhornia crassipes*. Given the decision-support orientation of this review, such operational sources represent a particularly valuable avenue for future synthesis, and their systematic incorporation would help bridge the gap between published methodology and field practice. In addition, the strong heterogeneity across studies in terms of sensor configurations, spatial and temporal resolutions, classification schemes, and validation metrics (e.g., overall accuracy, F1 score, intersection over union) limited the possibility of conducting a formal quantitative meta-analysis. As a result, the review relies on interpretation rather than statistically standardized comparison, which constrains the ability to derive generalized performance benchmarks across platforms and methods. Furthermore, many studies report accuracy metrics based on limited or non-independent validation datasets, potentially leading to optimistic performance estimates and reducing the robustness and comparability of reported results across different environmental contexts.

A second set of limitations arises from methodological and operational gaps identified in the reviewed literature. Although multi-sensor and multi-platform integration is increasingly proposed as a key strategy for invasive plants monitoring, most studies remain site-specific and lack an explicit evaluation of model transferability across different ecosystems, seasons, and water quality conditions. This limits the generalization of classification models, particularly in heterogeneous environments where spectral mixing, turbidity, sun glint, and phenological variability significantly affect detection performance. Additionally, while this review proposes a decision-support framework, relatively few studies explicitly link remote sensing outputs to actionable management thresholds, economic constraints, or intervention strategies, highlighting a persistent gap between methodological development and operational implementation. Finally, the rapid evolution of remote sensing technologies and machine learning approaches means that recent advances, particularly in deep learning and near-real-time monitoring systems, may not yet be fully represented in the literature. Despite these limitations, this review provides a robust synthesis of current methodologies and identifies critical directions for advancing the operational use of remote sensing in the monitoring and management of *Eichhornia crassipes.*

## Conclusion

As aquatic ecosystems face increasing pressure from biological invasions, climate variability, and anthropogenic nutrient enrichment, *Eichhornia crassipes* has emerged not only as a persistent management challenge but also as a valuable indicator of ecological disruption. This review synthesizes the growing body of research that leverages remote sensing to detect, classify, and assess the impacts of *E. crassipes* across spatial and temporal scales. We demonstrate that meaningful progress has been made in multi-sensor data integration, species-level classification, and the development of indices capable of capturing biomass and physiological stress. However, significant opportunities remain to improve the predictive capacity of remote sensing systems, particularly through time-series modeling, early-warning indicator development, and the systematic inclusion of phenological and ecological context in classification frameworks.

Looking ahead, remote sensing must evolve from descriptive mapping to integrated decision-support systems for monitoring and managing *Eichhornia crassipes*. This requires continued refinement of scalable analytical pipelines, the incorporation of spectral markers for pollutant detection and phenological stress, and the formal integration of sensing outputs into adaptive management workflows. With advances in UAV platforms, hyperspectral sensors, and machine learning algorithms, there is a clear pathway toward operational systems that are responsive, interpretable, and transferable across sites and seasons. Future efforts should emphasize cross-platform calibr=ation, open-access data pipelines, and co-development with local stakeholders to ensure that technological advances translate into ecological insight and practical management outcomes. When embedded in this broader framework, remote sensing can support early detection, spatial prioritization, intervention planning, and post-control evaluation, thereby playing a pivotal role in the sustainable management of *Eichhornia crassipes* across diverse freshwater systems.

## Data Availability

No datasets were generated or analyzed during the current study.
